# Branch Retinal Artery Occlusion after Percutaneous Coronary Intervention

**DOI:** 10.1155/2021/9985568

**Published:** 2021-04-27

**Authors:** Tommaso La Macchia, Remo Albiero, Tommaso Invernizzi, Giorgia Ceravolo, Ida Ceravolo

**Affiliations:** ^1^Department of Clinical and Experimental Medicine, Unit of Cardiology, University of Messina, Via C. Valeria, 98125 Messina, Italy; ^2^Interventional Cardiology Unit-Sondrio Hospital, Via Stelvio, 25, 23100 Sondrio, Italy; ^3^Ophthalmology Unit-Sondrio Hospital, Via Stelvio, 25, 23100 Sondrio, Italy; ^4^Department of Human Pathology and Evolutive Age “Gaetano Barresi”, University of Messina, Via C. Valeria, 98125 Messina, Italy; ^5^Department of Clinical and Experimental Medicine, University of Messina, Via C. Valeria, 98125 Messina, Italy

## Abstract

We report a case of branch retinal artery occlusion (BRAO) that occurred after percutaneous coronary intervention (PCI). A 59-year-old man with no other previous diseases presented visual acuity deterioration in the left eye 24 hours after PCI. Fundus examination revealed ischemia at the temporal branch of the retinal artery associated with inner layer edema. Prompt treatment was performed with ocular digital massage and paracentesis of the anterior chamber. However, at discharge, the patient had a persistent visual loss with a central scotoma that persisted at 35-day follow-up without improvement of the visual acuity. The patient did not suffer from any other systemic complications. Retinal infarction should be considered a potential complication of PCI. Patients and health care providers should be aware of any visual signs. Permanent visual disability can be prevented by immediate diagnosis and prompt intervention.

## 1. Introduction

Percutaneous coronary intervention (PCI) is currently indicated for the management of patients presenting with coronary artery disease (CAD). Advances in technology, development of more potent and effective antiplatelet therapy, and judicious use of PCI are increasing the safety of the procedure. However, major complications during and after PCI still occur that can be related to the access site, intubation of the coronary artery ostia, or the intervention itself. The most common problems occur in the context of coronary intubation and target vessel or site intervention [1]. However, different organs including renal, brain, eye, and gastrointestinal systems could be targets of thromboembolic events during PCI or cardiac catheterization [2]. In particular, the association with some retinal alterations has been occasionally reported [3–9]. We report a case of branch retinal artery occlusion (BRAO) that occurred 24 hours after PCI.

## 2. Case Report

In November 2020, a 59-year-old man, an ex-smoker with negative previous medical history of heart disease, was admitted to the emergency department of Sondrio Hospital (Sondrio, Italy), 48 hours after the onset of episodes of rest angina associated with atypical angina symptoms (jaw pain in the dental arches).

**Figure 1:**
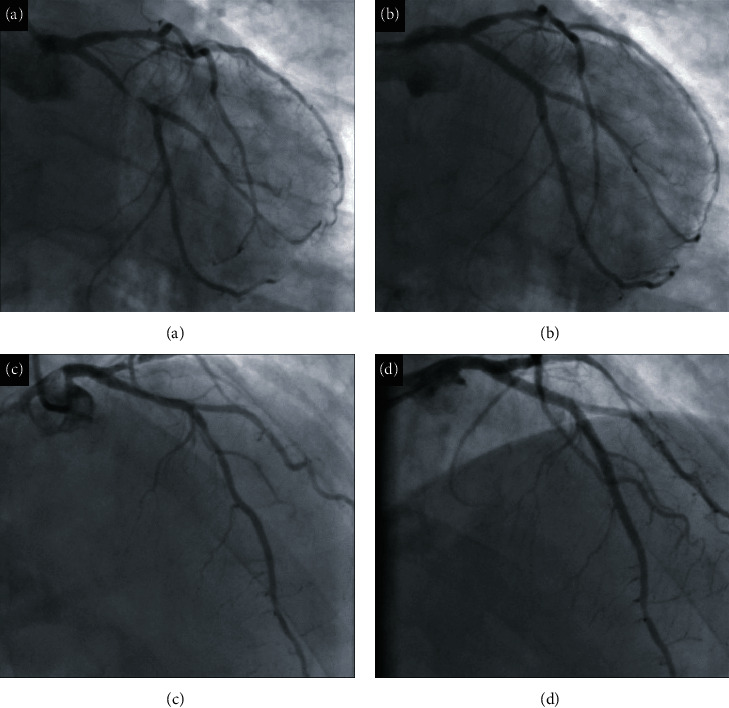
The angiogram shows 2-vessel CAD (coronary artery disease) with subocclusion of the proximal circumflex coronary artery (LCx) (a) and critical stenosis of the proximal and middle anterior descending coronary artery (LAD) (c). Good final result after PCI with implantation of a DES (drug-eluting stent) in the proximal-middle LCx at the bifurcation with the MO1 branch (b) and two contiguous DES on the proximal-middle LAD (d).

Admission ECG showed a heart rate of 95 bpm and a slight ST segment depression in the lateral leads. Measurement of cardiac biomarkers showed a rise in high-sensitivity troponin I (hs-cTnI, 142 ng/L). Double antiplatelet therapy (DAPT) with aspirin 100 mg/day and clopidogrel 75 mg/day was started on admission, and the same day, the patient underwent coronary angiography by right transradial access. The angiogram showed 2-vessel CAD (coronary artery disease) with subocclusion of the proximal circumflex coronary artery (LCx) and critical stenosis of the proximal and middle anterior descending coronary artery (LAD) (Figures 1(a) and 1(c)). After administration of heparin 100 U/kg, ad hoc PCI was performed with implantation of a drug-eluting stent (DES) in the proximal-middle LCx at the bifurcation with the MO1 branch and two contiguous stents on the proximal-middle LAD, with a good final angiographic result on both vessels (Figures 1(b) and 1(d)).

**Figure 2:**
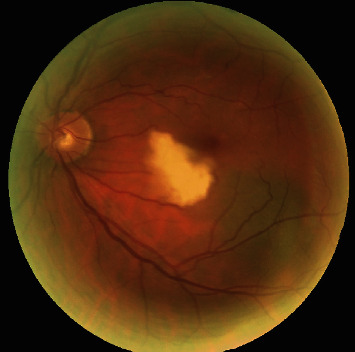
Fundus photograph of the left eye branch retinal artery occlusion (BRAO) showing white pallor at the inferior temporal branch of the central retinal artery.

**Figure 3:**
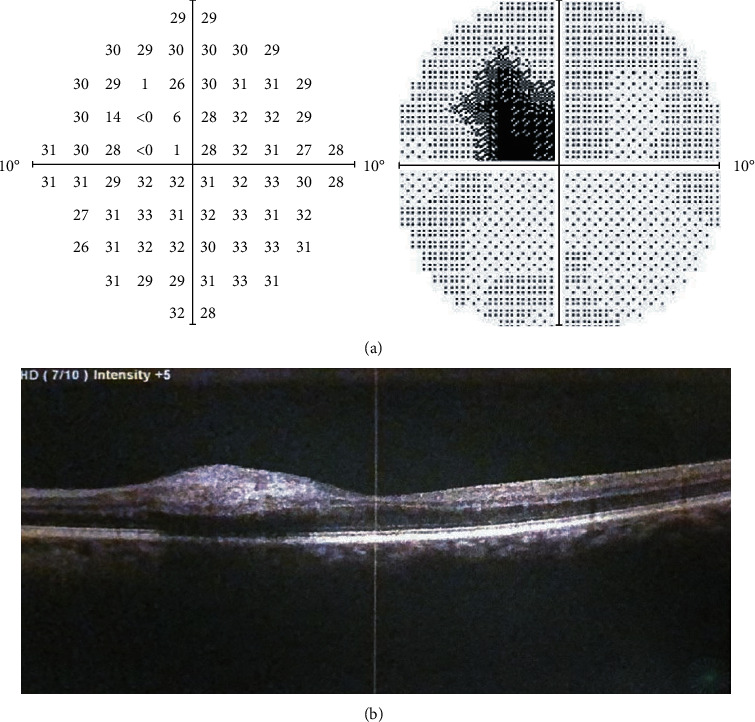
(a) Visual field examination demonstrating defects in the 5° temporal in the left eye. (b) Optical coherence tomography (OCT) images of the left eye showing temporal inner layer edema.

Twenty-four hours after PCI, the patient reported the onset of a left central scotoma and an ophthalmological evaluation was requested. The left visual acuity was reduced to 0.7 logMAR with a central scotoma. There was no alteration in the anterior segment. Intraocular pressure (IOP) was 10 mmHg. Fundus examination revealed a white embolus in the temporal branch of the central retinal artery (Figure 2). A computerized visual field (Humphrey II 740 Visual Field, Carl Zeiss Meditec AG, Jena, Germany) revealed a paracentral visual defect (Figure 3(a)). Moreover, spectral domain-optical coherence tomography (SD-OCT) (RS-3000 Nidek, Gamagori, Japan) was performed which showed inner layer edema in the temporal sector (Figure 3(b)). The right eye was normal. The diagnosis was consistent with BRAO. Immediate conventional treatment of BRAO was performed, with ocular digital massage and paracentesis of the anterior chamber. Complete neurological examination was normal. The patient underwent supra-aortic trunks echo-color Doppler (TSA) which showed bilaterally diffuse hyperplasia affecting the common carotid, the carotid bulb, and the internal and external carotid in the absence of hemodynamically significant flow accelerations. It showed no morphological alterations of the supra-aortic vessels. No other systemic complications occurred during hospitalization. At discharge, the patient had a persistent visual loss with a central scotoma that persisted at 35-day follow-up. In addition, follow-up OCT showed persistent macular edema. This study followed the tenets of the Declaration of Helsinki, and written consent was obtained from the patient.

## 3. Discussion

Branch retinal artery occlusion (BRAO) is one common cause of retinal vascular disease; it is caused by a reduction in arterial blood flow, resulting in retinal ischemia which can lead to severe vision loss and visual field defects despite therapy. Commonly, it is associated with systemic risk factors including hypertension, diabetes, carotid artery disease, and atherosclerosis. Few studies suggest that different retinal complications could be observed after coronary angiography procedures. Eye involvement includes central retinal artery occlusion (CRAO) [3, 4], branch retinal artery occlusion (BRAO) [5–7], or cilioretinal artery occlusion [8]. In addition, retinal embolisms following coronary catheterization can be clinically silent with any visual impairment. Moreover, it has been found that the risk factor for retinal emboli can be significantly associated with patient age, hypertension, and operator expertise [9]. Our patient had no additional risk factors. In the current case, the concomitance between the onset of eye disease and the coronary procedure suggests a correlation between the two conditions.

An immediate treatment is required if the occlusion occurs within 24 hours from the procedure. Conventional therapy consists in the reduction of intraocular pressure with hypotensive drugs, digital massage of the eyelid, and anterior chamber paracentesis. In some cases, thrombolytics can be infused into the carotid artery, or a laser embolectomy can be performed. The goal of the treatment is reperfusion of the blocked retinal vessels particularly when it is done within 6 h, because retinal ischemic damage after 240 min is normally permanent [10]. However, treatments rarely improve visual acuity, and in BRAO, the visual field damage is typically permanent. Furthermore, in rare case, the condition could be complicated with retinal or iris neovascularization leading to glaucoma or vitreous hemorrhage.

## 4. Conclusion

With the growing occurrence of PCI, all physicians involved in patient care should be worried in keeping special attention towards ocular signs. Visual symptoms and retinal artery occlusions caused by embolization in patients after PCI could occur even 24 hours after catheterization. Prompt diagnosis and early treatment may help improve the outcome and preserve vision deterioration.

## Data Availability

No data were used to support this study.

## Additional Points

*Learning Objective*. Retinal infarction should be considered a potential complication of PCI with the risk of permanent visual disability that can be prevented by immediate diagnosis and prompt intervention. Patients and health care providers should be aware of any visual signs after PCI.
